# Implementing an Inclusive, Multidisciplinary Supportive Care Model to Provide Integrated Care to Breast and Gynaecological Cancer Survivors: A Case Study at an Asian Ambulatory Cancer Centre

**DOI:** 10.5334/ijic.6480

**Published:** 2023-03-17

**Authors:** Yu Ke, Yung Ying Tan, Patricia Soek Hui Neo, Grace Meijuan Yang, Kiley Wei-Jen Loh, Shirlynn Ho, Yee Pin Tan, Mothi Babu Ramalingam, Daniel Song Chiek Quah, Lita Chew, Phebe En Hui Si, Beng Choo Tay, Alexandre Chan

**Affiliations:** 1 Division of Supportive and Palliative Care, National Cancer Centre Singapore, SG; 2 Duke-NUS Medical School Singapore, SG; 3 Department of General Medicine, Sengkang General Hospital, SG; 4 Department of Psychosocial Oncology, National Cancer Centre Singapore, SG; 5 Department of Rehabilitation Medicine, Singapore General Hospital, SG; 6 Division of Radiation Oncology, National Cancer Centre Singapore, SG; 7 Department of Pharmacy, National University of Singapore, SG; 8 Department of Pharmacy, National Cancer Centre Singapore, SG; 9 Department of Clinical Pharmacy Practice, University of California Irvine, US

**Keywords:** distress screening, cancer, integrated care, supportive care, inclusive, COVID-19

## Abstract

**Introduction:**

Supportive care models considering inclusivity and community services to improve integrated care for cancer survivors are limited. In this case study, we described the implementation of a multidisciplinary care model employing routine distress screening and embedded integrated care pathways to integrate care across disciplines and care sectors, while remaining inclusive of the multi-ethnic and multilingual population in Singapore. We reported implementation outcomes after 18 months of implementation.

**Description:**

We reviewed the model’s process indicators from September 2019 to February 2021 at the largest public ambulatory cancer centre. Outcomes assessed included penetration, fidelity to screening protocol, and feasibility in three aspects – inclusiveness of different ethnic and language groups, responsiveness to survivors reporting high distress, and types of community service referrals.

**Discussion/conclusion:**

We elucidated opportunities to promote access to community services and inclusivity. Integration of community services from tertiary settings should be systematic through mutually beneficial educational and outreach initiatives, complemented by their inclusion in integrated care pathways to encourage systematic referrals and care coordination. A hybrid approach to service delivery is crucial in ensuring inclusivity while providing flexibility towards external changes such as the COVID-19 pandemic. Future work should explore using telehealth to bolster inclusiveness and advance community care integration.

## Introduction

According to the National Cancer Institute, a person with a cancer diagnosis is termed a survivor from diagnosis to the end of life [1]. Supportive care models aim to promote integrated care provision to survivors through the delivery of supportive care services, such as psycho-oncology services, rehabilitation, and palliative care, to prevent and manage adverse effects from cancer and its treatment [2,3]. Among cancer survivors, an integrative approach combining different health disciplines is necessary to effectively manage the myriad of physical, emotional, social, psychological, practical, and spiritual issues throughout survivorship [4,5].

Typically, cancer survivors are managed under the oncologist-led model where oncologists serve as the predominant cancer-related care providers in the tertiary setting [6]. Hence, oncologists play the pivotal role in providing supportive care and initiating referrals to supportive care services [3]. Two major gaps exist in this oncologist-centric model that warrants a paradigm shift. First, there is a disproportionate focus on managing physical problems than psychosocial issues due to clinical time constraints, inadequate education and training, a lack of awareness of available services, and a lack of knowledge about how to integrate attention to psychosocial health needs into their practices [7,8,9,10]. Second, integrated care provision across disciplines and sectors is compromised with inadequate infrastructural support and a lack of clearly defined referral pathways available for oncologists [8,11]. Consequently, cancer survivors’ access to supportive care services is heterogenous, fragmented, and subjected to significant inter-oncologist variability [7]. Care fragmentation is further associated with reduced resource allocation efficiency, poorer symptom control, higher costs, and failure to deliver consistent and coordinated patient-centred care [12,13].

Supportive care models employing routine distress or care needs screening with follow-up care are then increasingly advocated and implemented to allow a holistic assessment of cancer survivors’ needs for survivor-centric care personalization [14,15,16,17]. The basis for the theoretical benefits of such models includes early identification, evaluation, and timely management of active problems. Recognizing the diversity of care needs across the survivorship continuum, these models tailor evidence-based interventions and coordinate multidisciplinary supportive services.

However, several knowledge gaps remain to be addressed. First, existing evidence on the effectiveness of such supportive care models are inconclusive due to poor reporting of implementation outcomes [18]. Second, integrated care provision needs to stay inclusive of survivors across different cultural backgrounds, languages, and health literacy levels to avoid potential health disparities [19,20]. However, there is limited literature describing inclusivity considerations during care design and inclusiveness is rarely evaluated as an outcome measure. Lastly, while there are increasing calls for community engagement to provide integrated cancer survivorship care across health sectors, inadequate cancer-related knowledge among community care providers and suboptimal care coordination across settings continue to pose as challenges [21]. In summary, there is a demand for robust evaluation of supportive care models implemented with considerations of inclusivity and access to community services to improve integrated care for cancer survivors.

Singapore is a high-resource country in Southeast Asia with a multi-ethnic and multilingual population. The design and launch of the Accessible Cancer Care to Enable Support for Cancer Survivors (ACCESS) supportive care model in National Cancer Centre Singapore (NCCS) was intended to address the abovementioned knowledge gaps. The ACCESS model employed an inclusive design and routine distress screening to triage cancer survivors with differing care needs. Specifically, embedded integrated care pathways serve as facilitating mechanisms to integrate care referrals to different health disciplines in the tertiary setting and integrate survivorship care across tertiary and primary care sectors. This study aims to describe the ACCESS implementation process and to report implementation outcomes after an 18-month period.

## Methods

### Study design

This study is part of a pragmatic type I effectiveness-implementation hybrid study designed to evaluate the impact of the ACCESS model in an ongoing cluster randomized controlled trial (NCT04014309) while collecting information on its implementation [22]. The literature recommended this hybrid approach to collect both implementation and effectiveness data to develop an evidence base that could promote widespread intervention adoption [23].

We focused on reporting the implementation outcomes from September 2019 to February 2021 here. This implementation study was approved by the SingHealth Centralised Institutional Review Board (CIRB 2020/2789) with the waiver of informed consent. Reporting of implementation metrics followed the Standards for Reporting Implementation Studies, and the model was described according to the minimum recommended components [24,25].

### Setting and target population

NCCS is the largest comprehensive ambulatory cancer centre serving 65% of adult cancer patients in Singapore’s public sector [26]. Funding from Temasek Foundation Cares supported the scale of model implementation in half of the medical oncologist clinics seeing breast and gynaecological cancer survivors, the most common incident cancers in women locally [27,28]. Cancer survivors were eligible if they were ≥21 years, diagnosed with breast or gynaecological cancer, and had received outpatient care from participating medical oncologists minimally once during the review period.

### Theoretical basis of the ACCESS model

**Figure 1:**
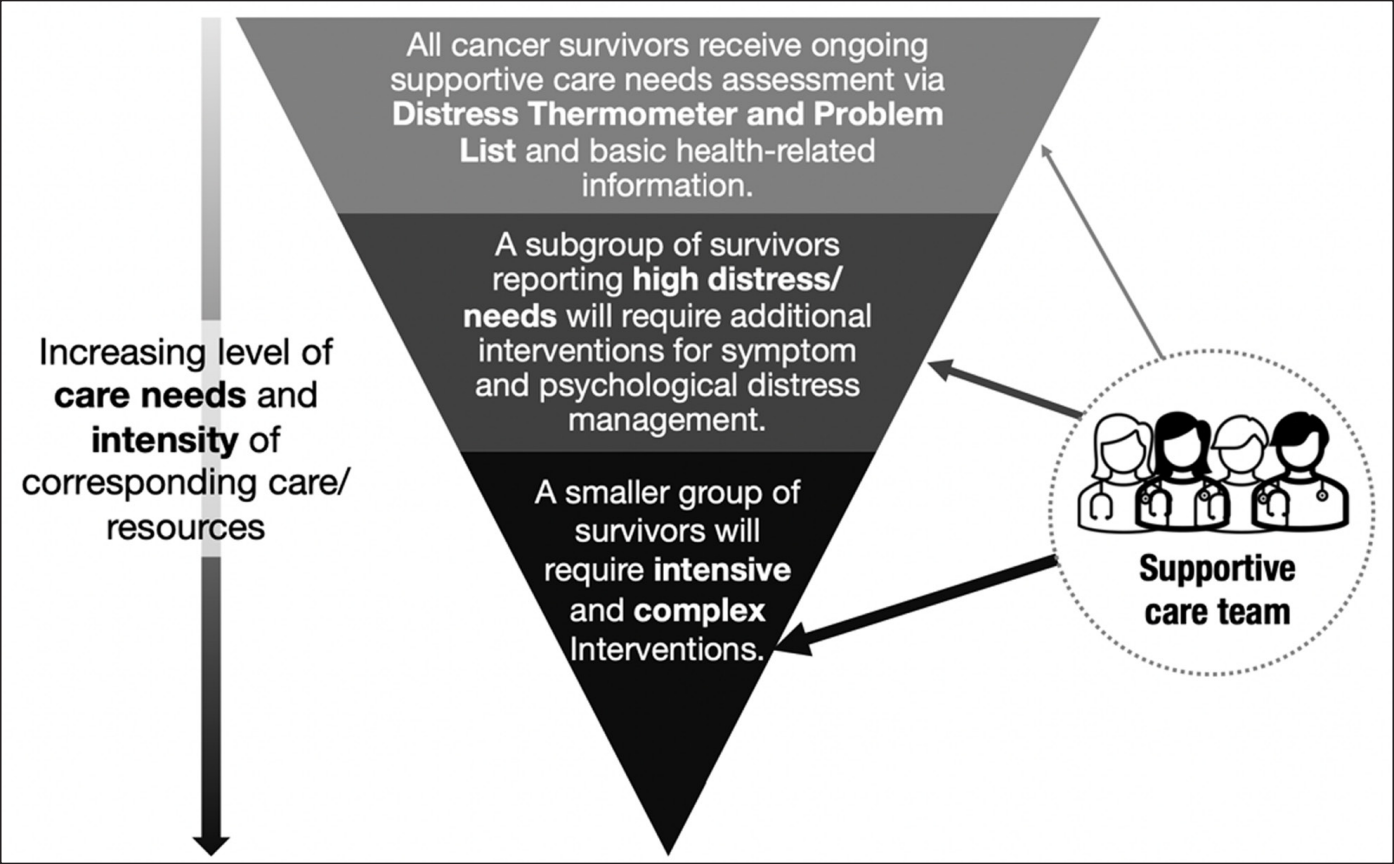
Tiered care approach of the Accessible Cancer Care to Enable Support for Cancer Survivors model. Routine distress and care needs screening were provided to all cancer survivors before triaging to further follow-up by a supportive care team based on reported levels of care needs.

The ACCESS model adopted the supportive care framework outlined by Fitch et al. (Figure 1) that described a tiered approach with two underlying principles [4,17]. First, basic health information and distress and care needs screening were offered to cancer survivors at all medical oncologist visits, regardless of language preference, and literacy levels. Second, access to supportive care resources was then determined based on the level of distress and care needs.

### ACCESS model implementation design and strategies

Implementation of the ACCESS intervention was guided by the Consolidated Framework for Implementation, which outlined determinants of successful implementation in the following domains: individual survivor characteristics, intervention characteristics, processes, and the organizational setting [29]. Complementarily, implementation strategies were developed to maximize screening outreach, facilitate the timely identification of survivors with high care needs, and improve access to tertiary and community support services. Implementation strategies are methods or techniques used to enhance the adoption, implementation, and sustainability of a clinical program [30]. Table 1 details the employed implementation strategies according to reporting recommendations [30,31].

**Table 1:** Summary of implementation strategies employed in the Accessible Cancer Care to Enable Support for Cancer Survivors intervention.


STRATEGY	CFIR DOMAIN	ACTORS AND ACTIONS	TARGET AUDIENCE	TEMPORALITY	JUSTIFICATION	ANTICIPATED IMPACT

Establish a core workgroup to ensure procedural accountability	Organizational (inner setting)	The core workgroup met regularly to discuss implementation challenges and elicit feedback for workflow streamlining.	Core workgroup, clinicians delivering the intervention, service coordinators	Monthly meetings were held with key process indicators reporting to the funder every quarter.	Facilitates iterative feedback and improvement process and brainstorms strategic engagement with stakeholders.	Enhanced feasibility and sustained intervention adoption.

Develop educational initiatives	Process	The core workgroup collaborated with the institutional education department to develop informational resources and plan educational activities.	Cancer survivors and caregivers, community service partners	Educational activities were concurrent with intervention implementation.	Promotes awareness of intervention and self-management by survivors and network weaving with community partners through educational outreach efforts.	Improved adherence to screening, increased number of trained community partners, and increased referrals to community services.

Strengthen the inclusivity of screening procedures	Individual survivor characteristics, intervention characteristics, process	The core workgroup culturally adapted the screening tool for the local population and translated it into multiple languages. Informational system and trained service coordinators supported a hybrid screening administration mode.	Cancer survivors	Translation occurred before the intervention launch. Efforts to improve the informational system for EMR integration are ongoing.	Targets survivor-level language and health literacy barriers to enhance screening uptake and adherence.	No systematic differences in screening completion rates across survivors of different ethnicities and health literacy levels.

Develop standardized, integrated care pathways	Intervention characteristics, process	The core workgroup led the development of standardized care pathways to map evidence-based interventions to all reported problems in the screening tool.	Supportive care team members	Pathways were drafted before intervention launch and amenable to changes following workgroup meetings.	Reduces variation in clinical practice across supportive care team members and provides systematic access to appropriate service referrals.	High responsiveness to survivors reporting high distress levels.


Abbreviations: CFIR, Consolidated Framework for Implementation Research; EMR, electronic health records.

#### Core workgroup formation

A multidisciplinary core workgroup that included oncologists, a rehabilitation physician, nurses, clinical pharmacists, and a psychologist oversaw the ACCESS model implementation. The workgroup met monthly to review implementation progress and anchored outreach efforts to relevant stakeholders (NCCS institution leaders, participating oncologists, funder, and the Singapore Ministry of Health), actively addressing implementation issues and elucidating analyses required for funding considerations to support long-term implementation.

#### Education initiatives

Collaborating with the NCCS Cancer Education and Information Services and corporate communications, the workgroup revamped, consolidated, and standardized available education and community resources on the institution website [32]. A leaflet summarizing the ACCESS model and containing a link/QR code to this institution website was provided to all survivors to empower them and their caregivers to self-manage problems, promoting self-efficacy [33]. Furthermore, to engage community partners in cancer survivorship care delivery, the workgroup gathered expertise from multiple disciplines to plan symposiums and lead educational activities, including experiential clinic attachments and curriculum development. Through these outreach efforts, an extensive list of available community services was consistently updated and mapped to survivorship care needs, facilitating downstream systematic referrals based on survivors’ reported problems.

#### Inclusive screening procedures

**Figure 2:**
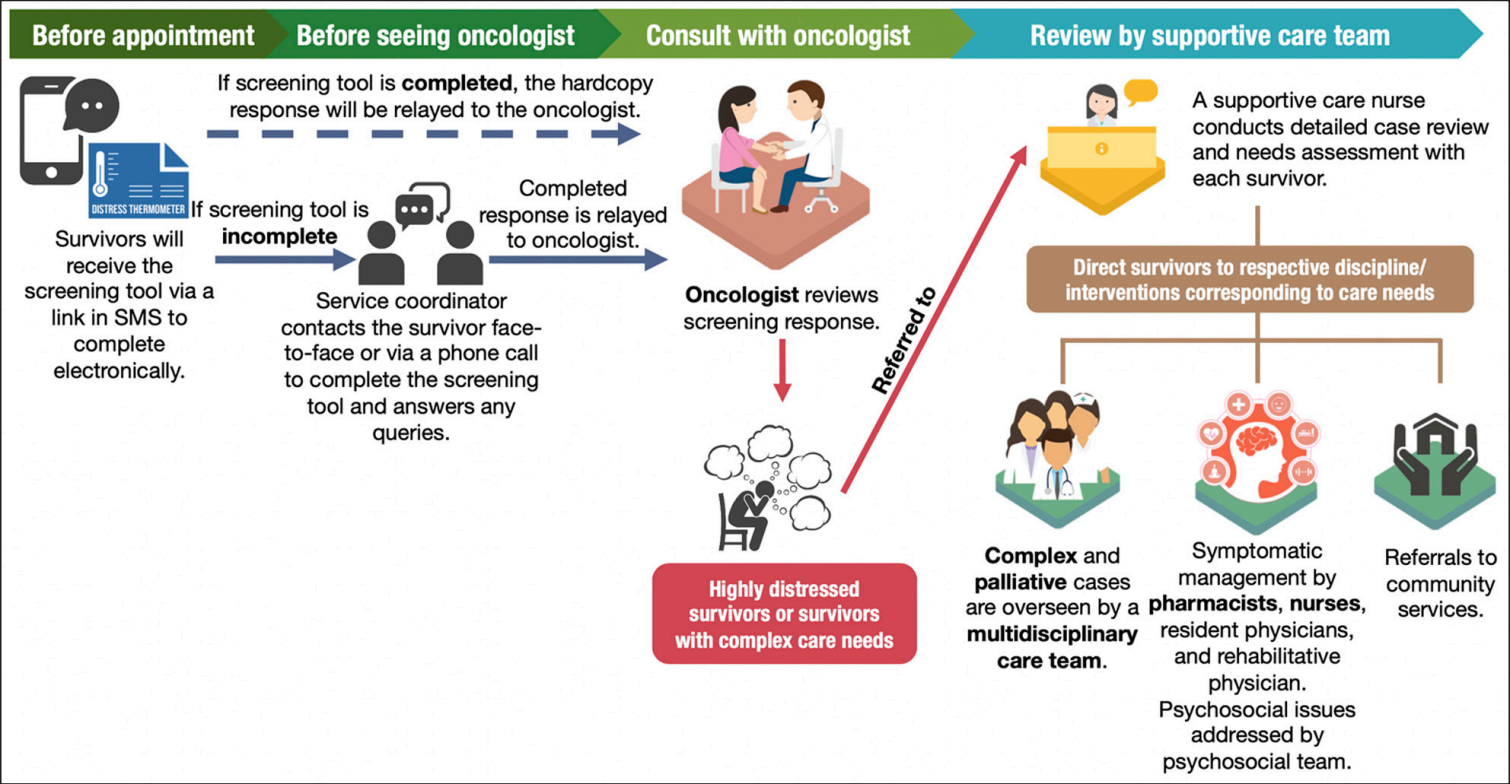
Overview of the clinical workflow for the screening procedures using the Distress Thermometer and Problem List, discussion during oncologists’ review, and subsequent management of survivors referred to the supportive care team.

**Figure 3:**
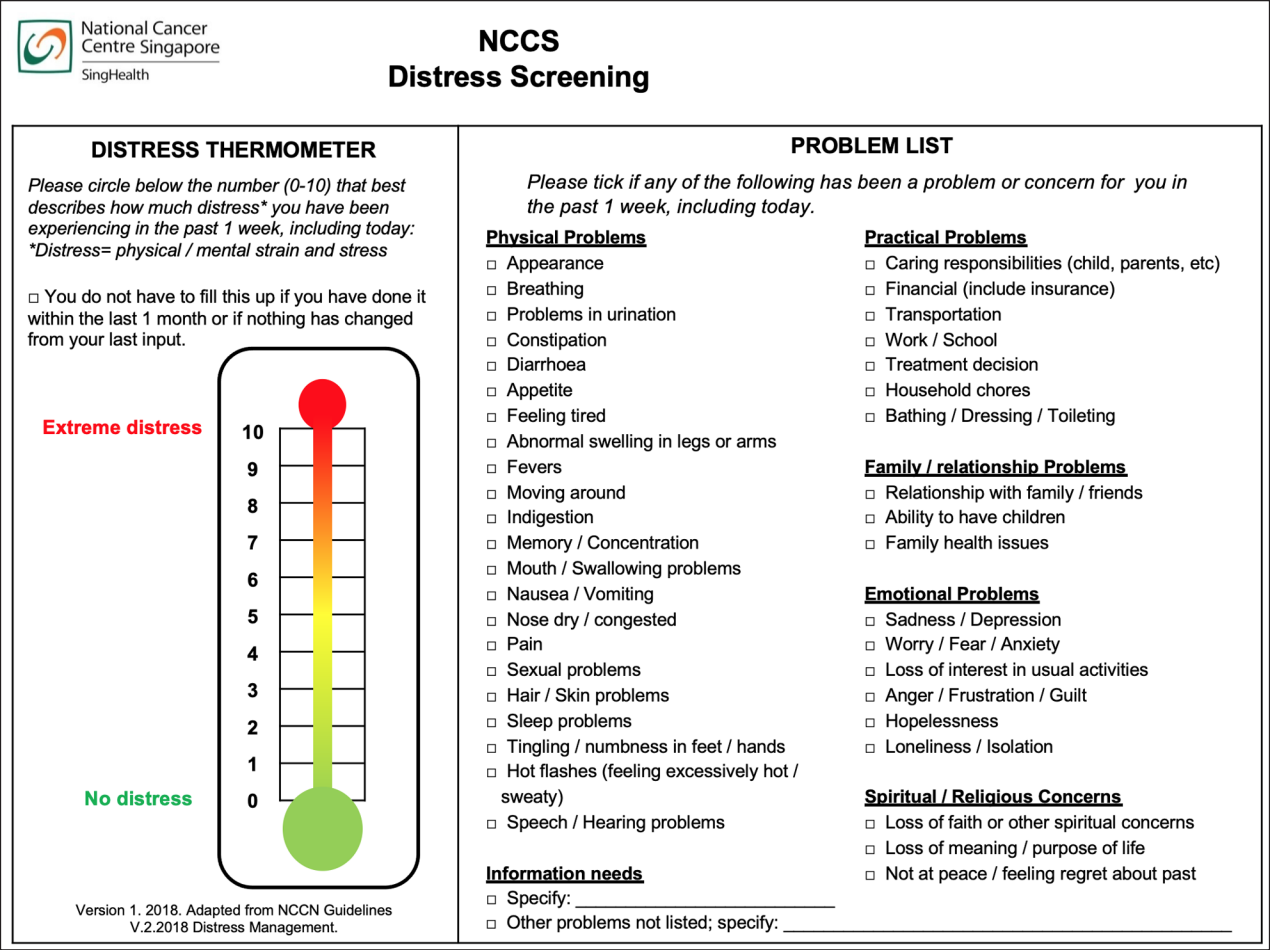
Distress Thermometer and Problem List adapted from the National Comprehensive Cancer Network.

Trained service coordinators with science-related degrees facilitated the screening process (Figure 2). The ACCESS model was introduced to all survivors at their first visit during the review period. Subsequently, before each medical oncologist visit, survivors received unique webpage links to the screening tool (Distress Thermometer and Problem List, DTPL) via short messaging services. The original English version was culturally adapted from the National Comprehensive Cancer Network (Figure 3) [17]. Survivors rated their distress levels on a Likert scale of 0-10 and indicated the problems (physical, practical, family, emotional, and spiritual) experienced in the past week. Self-administration typically took 5-10 minutes and responses were captured electronically. Several strategies were adopted to maximize inclusivity. First, the DTPL was translated into other official languages of Singapore (Mandarin, Malay, or Tamil) to accommodate the multilingual population. Second, on the day of clinic visit, service coordinators either approached or contacted survivors who did not complete the screening tool electronically beforehand to administer the tool and address usability concerns. Lastly, hardcopy of the DTPL was also available for survivors without access to phones or internet.

#### Clinical responses to screening results (Figure 2)

All screening responses were physically relayed to oncologists for review and discussion during consultations. Survivors were referred to the supportive care team within the same day when (i) survivors reported a DT score ≥6, indicative of high distress, and/or (ii) additional review was deemed necessary based on oncologist’s discretion, regardless of DT score. A higher DT score of 7 was selected initially and revised to 6 after two months of implementation based on manpower capacity considerations. Both scores could identify participants with clinically significant distress in the local population [34]. All highly distressed survivors were offered a review with the supportive care team in-person or through telehealth.

#### Review by a multidisciplinary supportive care team using integrated care pathways (Figure 4)

**Figure 4:**
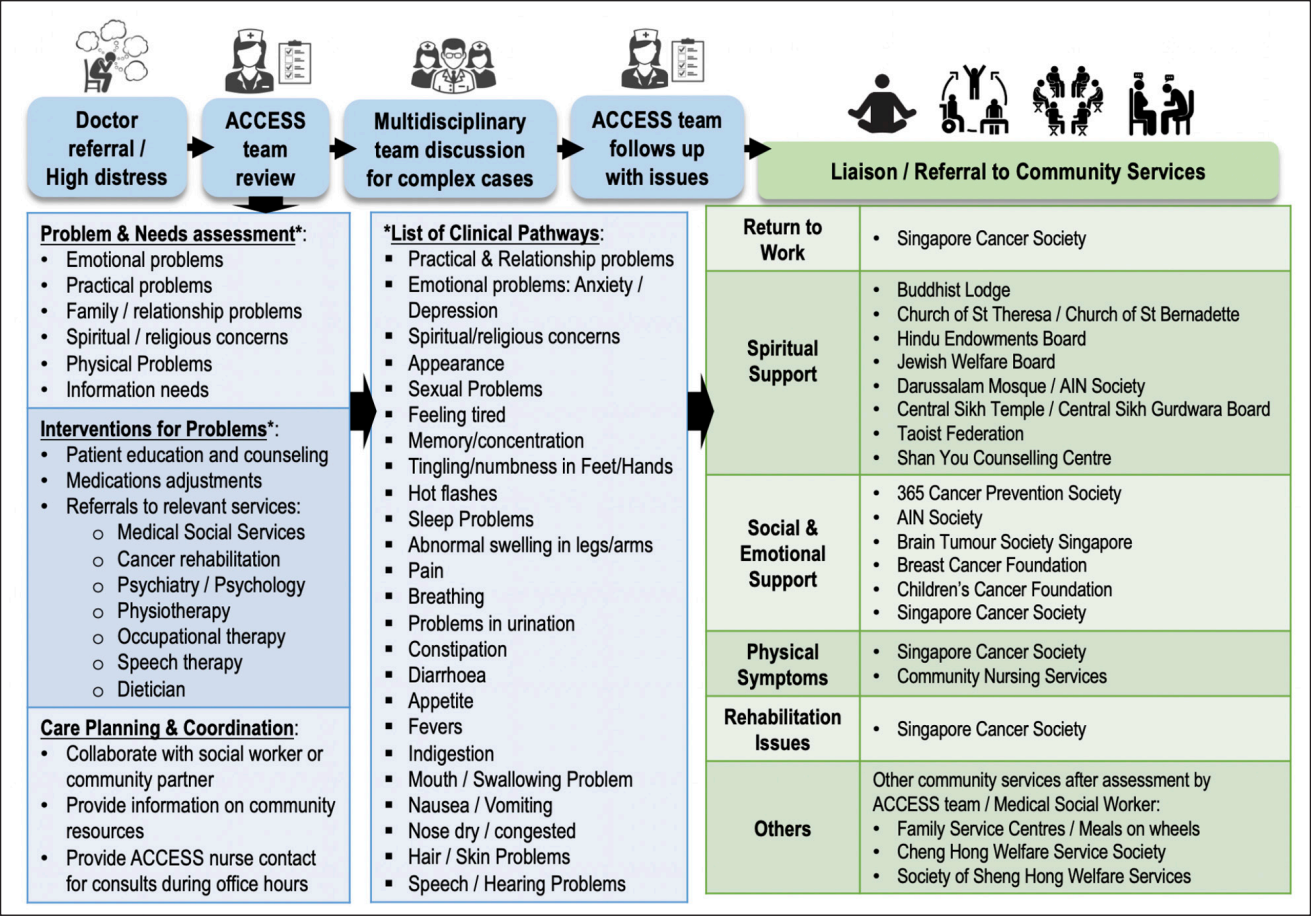
Overview of supportive care interventions and a network of community services referrals available for survivors based on care needs.

Typically, a supportive care nurse would first conduct detailed needs assessments for each referred survivor before providing care according to standardized integrated care pathways. These care pathways were available for all problems reflected on the DTPL to ensure that screening results lead to actionable interventions. The workgroup developed these pathways by locally adapting interventions and recommendations from guidelines to reduce clinical variation when addressing reported supportive care needs. The nurse then facilitated access to other team members from diverse health disciplines (e.g., pharmacy, psychology, social work, rehabilitation) and community services. A further subgroup of survivors who presented with complex needs or palliative concerns were referred to multidisciplinary care team biweekly reviews.

### Impact of COVID-19

Responding to COVID-19, Singapore raised its disease outbreak alert to orange in February 2020, enforcing control measures and mobility restrictions [35]. A lockdown was imposed from 7 April to 1 June 2020 before gradual reopening [36]. Workflows were modified to adhere to institutional guidelines during periods of tightened restrictions. First, non-emergency visits were postponed. Second, all in-person encounters between service coordinators and survivors were replaced by calls. Third, relaying of screening results to oncologists was impermissible during the lockdown. In contrast, supportive care team workflows were minimally disrupted as telehealth consult was already available.

### Outcome evaluation

We assessed the following implementation outcomes based on Procter et al.’s taxonomy [37]. We determined the penetration of our model by quantifying the number of survivors introduced to the ACCESS model and those who received self-help educational resources. Next, we assessed fidelity by tabulating adherence rates to the screening protocol based on the proportion of attended clinic visits with corresponding screening responses. Adherence rates were categorized into: <50%, reflecting adherence less than half of the time; 50–99%, reflecting adherence most of the time; and 100%, reflecting complete adherence.

The feasibility of implementing the model in real-world settings was assessed in three aspects. First, the inclusivity of the screening procedures was evaluated by determining whether screening tool completion rates differed by ethnicity and language preferences. The mode of screening tool completion was summarized as a proxy to measure the inclusiveness of survivors with lower literacy or who were less technology-savvy. Second, the proportion of screened survivors reporting high distress was determined. Responsiveness to these survivors was evaluated by tabulating the proportion of highly distressed survivors who received access to the supportive care team. Third, the access to community services was described by the number and types of referrals made to community services and corresponding acceptance and attendance rates. Lastly, implementation cost was assessed by the additional manpower required to support the screening procedures which were not part of usual care. Additional subgroup analyses were conducted to assess the impact of COVID-19 restrictions on penetration, mode of screening, and screening completion rates.

### Sample size calculation and statistical analysis

Based on past clinical load, we expected 3500 eligible survivors within the 18-month review period. All data were analysed using STATA 17. Descriptive statistics were used to describe survivors’ characteristics and outcome indicators. Mean and standard deviation were used to summarize continuous variables, while counts and percentages were used for categorical variables. A multivariable Poisson regression model was used to analyse screening tool completion rates by ethnicity and language preferences. The model was further adjusted for survivors’ age where adherence rates among the adolescent and young adult group were significantly lower than expected compared to older survivors in a study that evaluated distress screening outcomes across Commission on Cancer-accredited cancer programs [38]. All statistical tests were 2-sided with *α* =.05.

## Results

### Penetration

Among 2357 eligible breast and gynaecological cancer survivors, we successfully introduced the ACCESS model and screened 1853 (78.6%) survivors minimally once. Screened survivors had mean (SD) age of 60.7 (11.2) years, were predominantly Chinese (1496/1853, 80.7%) and were mostly diagnosed with breast cancer (1683/1853, 90.8%) (Table 2). Self-help educational resources were distributed to 1518 (81.9%) survivors.

**Table 2:** Characteristics of ACCESS model recipients (N = 1853).


CHARACTERISTIC	N (%)

Age, mean (SD)	60.7 (11.2)

Ethnicity	

Chinese	1496 (80.7%)

Malay	186 (10.0%)

Indian	115 (6.2%)

Others	56 (3.0%)

Cancer diagnosis	

Breast	1683 (90.8%)

Gynaecological	170 (9.2%)

Language preference for screening tool	

English	1444 (77.9%)

Mandarin	385 (20.8%)

Malay	21 (1.1%)

Tamil	3 (0.2%)


### Fidelity and implementation cost

Overall, the tasks and time required to support screening amounted to service coordinators operating at two full-time equivalents (Table 3). The overall adherence rate to the screening protocol was 77.4%, with 5779 screening responses obtained from 7464 visits. For 1259 survivors with multiple visits during the review period, 412 (32.7%) survivors completed the screening tool at between 50-99% of their visits, while 683 (54.3%) survivors demonstrated complete adherence (Table 4).

**Table 3:** A summary of tasks and time required to support screening procedures weekly based on an estimated caseload of 150 survivors per week.


TASK	TIME REQUIRED	FREQUENCY	TOTAL TIME REQUIRED PER WEEK (MINUTE)

Export a list of scheduled visits in the upcoming week	30 minutes for all patients	Weekly	30

Register survivors in the electronic system before DTPL could be disseminated	5 minutes per survivor	Weekly	750

Schedule DTPL for the electronic system to trigger a SMS with the link for completion to survivors a few days before scheduled visits	10 minutes per survivor	Weekly	1500

Download DTPL responses from the electronic system into a compiled Excel spreadsheet	5 minutes per download	Daily	25

Transpose compiled responses into individual responses	5 minutes per response	Daily	750

Relay screening results to oncologists in clinics before consults physically and assist survivors with in-person completion of screening tool	Full clinic hours (6 hours daily)	Daily	1800

**Total**	4855 (equivalent to approximately **2 FTE**)		


Abbreviations: DTPL, Distress Thermometer and Problem List; FTE, full-time equivalent.

**Table 4:** Screening completion rates among survivors with multiple medical oncology visits during the 18-month implementation period (N = 1259).


NUMBER OF VISITS IN THE REVIEW PERIOD	COMPLETION RATE		

	<50%, N (%)	50–99%, N (%)	100%, N (%)

All survivors with multiple visits	164 (13.0%)	412 (32.7%)	683 (54.3%)

3 visits (n = 171)	28 (16.4%)	46 (26.9%)	97 (56.7%)

4 visits (n = 132)	9 (6.8%)	55 (41.7%)	68 (51.5%)

5 visits (n = 119)	17 (14.3%)	35 (29.4%)	67 (56.3%)


### Feasibility – inclusivity

Most screening responses captured were completed by survivors electronically (67.6%) without assistance by service coordinators. The English version was most used while 22.1% preferred non-English version. Completion rates were not found to differ by language preferences, before and adjusting for age and ethnicity (Table 5). Among ethnic groups, the screening tool completion rate was 15% lower among Malays [adjusted rate ratio (95% CI): 0.85 (0.76, 0.95), P = 0.004] than in Chinese, after adjusting for age and language preference.

**Table 5:** Poisson regression analysis of screening completion rates for survivors with multiple medical oncology visits (N = 1259).


VARIABLES	UNADJUSTED COMPLETION RATE RATIO (95% CI)	ADJUSTED COMPLETION RATE RATIO (95% CI)

Ethnicity		

Chinese	Reference	Reference

Malay	0.89 (0.79, 0.99)	0.85 (0.76, 0.95)

Indian	1.01 (0.91, 1.12)	0.99 (0.90, 1.10)

Others	1.00 (0.87, 1.14)	0.98 (0.85, 1.14)

Preferred language		

English	Reference	Reference

Non-English	0.96 (0.90, 1.00)	0.95 (0.89, 1.02)

Age^a^	1.00 (0.99, 1.00)	1.05 (0.99, 1.00)


^a^ Modelled as a continuous variable.

### Feasibility – responsiveness

A total of 529/1853 (28.6%) screened survivors reported high distress minimally once during the review period. Among them, 231 (43.7%) survivors only reported high distress at repeat visits. Responding to this group, 19 (3.6%) survivors were uncontactable despite three separate call attempts on different days while attempts to contact another 89 (16.8%) survivors are ongoing. Eventually, 421(79.6%) survivors were contacted and reviewed by the supportive care team. After review, an additional 63/421 (15%) highly distressed survivors were referred to the multidisciplinary care team.

### Feasibility – access to community services

The supportive care team proposed community service referrals to 143/421 (34.0%) reviewed survivors, with 22 (5.2%) survivors receiving more than one referral (Table 6). Overall, the most common referral was to community hospice (69/143, 48.3%), followed by cancer-specific community services (28/143, 19.6%), primary care physicians (27/143, 18.9%), and community facilities (18/143, 12.6%). Acceptance rates (Table 7) of these four common referrals all exceeded 50%, with the highest acceptance rates for hospice (81.2%) and primary care physicians (81.5%). Attendance rates to accepted referral visits were the highest for hospice (79.3%) and community facilities (83.3%). A lower attendance rate for the remaining two services was accompanied by a higher proportion of referrals with visits planned after the study period or with untraceable outcomes.

**Table 6:** Type and frequency of community service referrals proposed to survivors reporting high distress (N = 529).


COMMUNITY SERVICE	PROPORTION OF HIGHLY DISTRESSED SURVIVORS WHO RECEIVED COMMUNITY REFERRALS BY THE SUPPORTIVE CARE TEAM, N (%)

Hospice	69 (13.0%)

Cancer-specific services^a^	28 (5.3%)

Primary care physicians	27 (5.1%)

Community facilities^b^	18 (3.4%)

Social services^c^	9 (1.7%)

Home-based care services	8 (1.5%)

Transport-related services	4 (0.8%)

Exercise programs	3 (0.6%)

Others^d^	6 (1.1%)


^a^ Includes 365 Cancer Prevention Society, AIN Society, Breast Cancer Foundation, Children’s Cancer Foundation, Singapore Cancer Society. ^b^ Includes community day care facilities, community nursing, rehabilitative centres. ^c^ Includes general, family, financial. ^d^ Includes eldercare, Ambulance Wish, Brave Charismatic, CGH Neighbours, private acupuncture, specialized disabled centre.

**Table 7:** Acceptance rates of community service referrals and subsequent attendance rates to accepted referral visits.


COMMUNITY SERVICE	ACCEPTANCE, N (%)	ATTENDANCE, N (%)

Hospice (n = 69)		

Referred/attended	58 (81.2%)	46 (79.3%)

Declined	11 (15.9%)	9 (15.5%)

Unknown/pending^a^	0 (0%)	3 (5.2%)

Cancer-specific services^b^ (n = 28)		

Referred/attended	17 (60.7%)	11 (64.7%)

Declined	7 (25.0%)	1 (5.9%)

Unknown/pending^a^	4 (14.3%)	5 (29.4%)

Primary care physicians (n = 27)		

Referred/attended	22 (81.5%)	10 (45.5%)

Declined	3 (11.1%)	3 (13.6%)

Unknown/pending^a^	2 (7.4%)	9 (40.9%)

Community facilities^c^ (n = 18)		

Referred/attended	12 (66.7%)	10 (83.3%)

Declined	6 (33.3%)	0 (0%)

Unknown/pending^a^	0 (0%)	2 (16.7%)


^a^ Refers to scheduled community visit that occurs after the study review period or the outcome of the community visit could not be traced. ^b^ Includes 365 Cancer Prevention Society, AIN Society, Breast Cancer Foundation, Children’s Cancer Foundation, Singapore Cancer Society. ^c^ Includes community day care facilities, community nursing, rehabilitative centres.

### Impact of COVID-19

**Figure 5:**
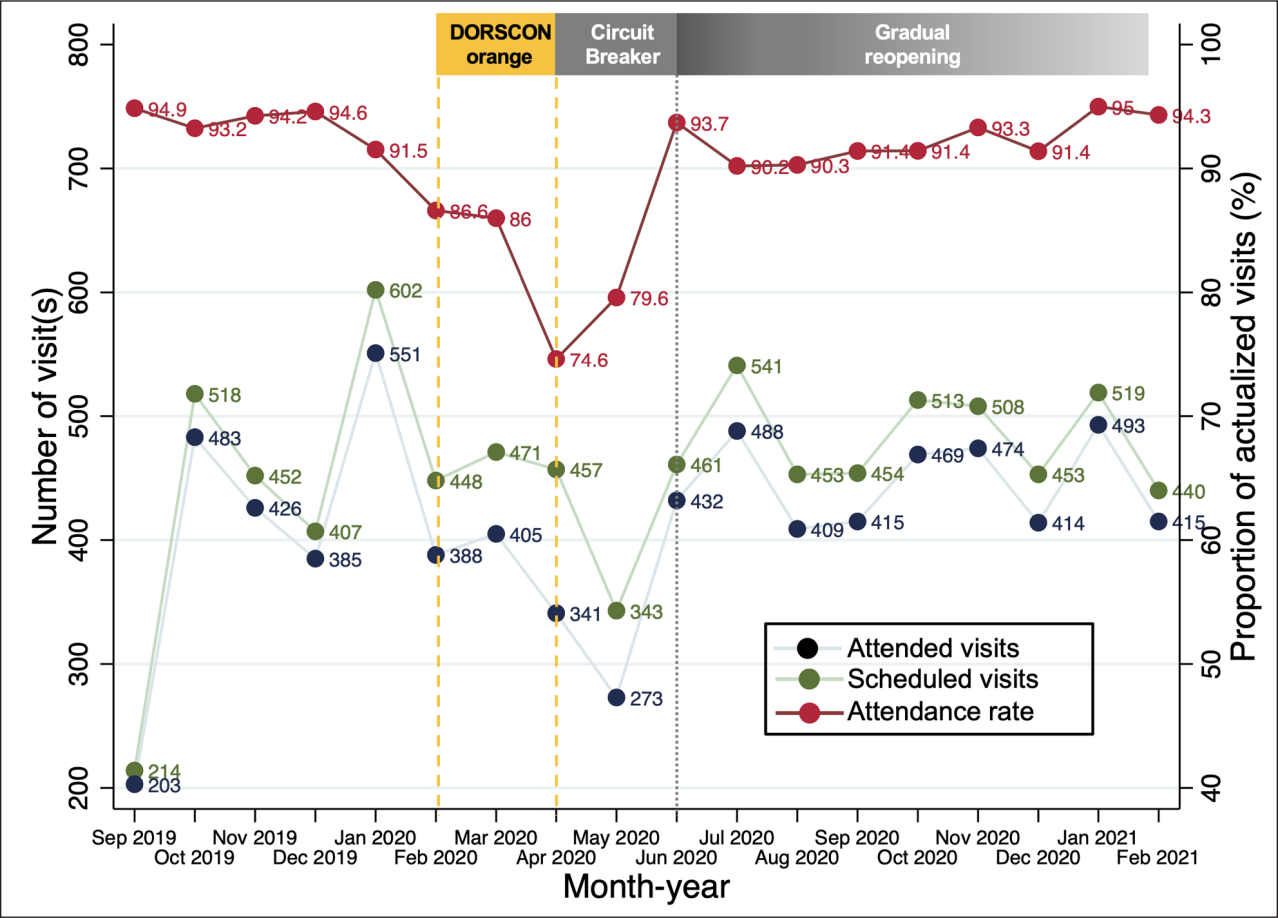
Number of scheduled and attended visits, and proportion of attended visits over the 18-month implementation period when significant COVID-19 restrictions were imposed. (DORSCON = Disease Outbreak Response System Condition, with orange representing severe disease and tightened control measures; Circuit Breaker = lockdown).

Both scheduled and attended visits were negatively affected by COVID-19, with a trough corresponding to the lockdown (Figure 5). Model penetration was reduced between February to June 2020 as the number of clinic visits dropped to <400. Caseload recovered to the average monthly level of 500 visits after gradual reopening, comparable with pre-COVID-19. Examining adherence to screening tool completion at different periods (Table 8), adherence rate decreased from 81.1% pre-COVID-19 to 64.6% during tightened measures, before returning to pre-COVID-19 levels (79.2%) after gradual reopening.

**Table 8:** Screening completion rates at attended medical oncologist visits stratified by periods of significant COVID-19 events.


OUTCOME	SIGNIFICANT COVID-19 EVENTS^a^			

	PRE-COVID-19 PERIOD	DORSCON ORANGE	CIRCUIT BREAKER	GRADUAL REOPENING

Attended visits, n (%)	2048 (93.4%)	793 (86.3%)	614 (76.8%)	4009 (92.3%)

Attended visits with screening tool completion, n (%)	1660 (81.1%)	512 (64.6%)	434 (70.7%)	3173 (79.2%)


^a^ Defined by the following time periods: pre-COVID-19 lasted from September 2019 to end January 2020, DORSCON Orange with social distancing and mobility restriction measures lasted from February to end March 2020, Circuit breaker (lockdown) lasted from April to early June 2020, gradual reopening phase refers to the remaining time periods. Abbreviation: COVID-19, coronavirus 2019; DORSCON, Disease Outbreak Response System Condition.

## Discussion

We presented results from the 18-month review of the ACCESS model which was successfully introduced to 1853 breast and gynaecological cancer survivors at an Asian ambulatory cancer centre in Singapore. Our findings demonstrated that a multidisciplinary care model like ACCESS, which employed routine screening with subsequent tiered care support using integrated care pathways, is feasible for incorporation into routine care. Additionally, our experience elucidated opportunities to improve referrals to community services and strategies to be inclusive of a diverse population in high-resource care settings to improve integrated cancer survivorship care provision.

Several studies evaluated the implementation of cancer supportive care models with distress screening following centre accreditation requirements and guidelines adherence [14,17]. Our screening protocol demonstrated satisfactory feasibility after successfully screening the majority (78.6%) of eligible survivors despite COVID-19, close to the upper bound of the 12-86% range reported in the literature [39,40,41]. Long-term adherence was exemplified by high fidelity to screening despite multiple visits, an important indicator as survivors may only report high distress at repeated visits. We drew strength from two crucial strategies that could extend across settings. First, regular engagement with involved personnel facilitated an iterative feedback and improvement process. Workflows were streamlined and clinical efficiency improved with increased experience and additional training support [42,43]. Second, a dedicated coordinator role served as a safety net to assist screening completion and guide survivors through the screening interface. This touchpoint reversed concerns of poor response rates [44,45] as the majority (67.6%) of responses were completed by survivors independently. Collectively, an engaged workforce improved the model’s feasibility and fidelity.

The ACCESS model identified 28.6% of screened survivors as highly distressed for supportive care team review, comparable to the 22-30% range reported in other outpatient cancer populations using DT cut-off score of 5-6 [46,47,48,49]. Screening allowed survivors to report emotional and practical problems, directly rectifying the current disproportionate focus on physical symptoms. Furthermore, our care team demonstrated high responsiveness to survivors’ care needs. Specifically, multidisciplinary care meetings ensured holistic support for survivors with complex needs who would not have received a comparable level of care intensity under usual care. Additionally, the supportive care team proposed a diverse range of community service referrals to 34% of survivors reporting high distress, promoting access to community services. Educational activities conducted for community partners increased awareness of available community services that could benefit survivors in the cancer centre. Although the absolute numbers of referrals to some services were low, we observed >50% acceptance and attendance rates to referral visits. Consistent with local and international calls for shifting survivorship care to community settings [21,50, 51], the ACCESS model epitomized a systematic approach to engage community services from tertiary settings through mutually beneficial educational and outreach initiatives.

Our experience demonstrated the impact of integrating inclusivity into the design of supportive care models. Inclusivity at the screening stage is pivotal in ensuring that all survivors have an avenue to report care needs. Echoing the need for improved care accessibility in underserved populations, our results revealed the ACCESS model’s inclusiveness of the multi-ethnic and multilingual Singapore population [19]. The ethnic distribution of our survivor cohort mirrored the distribution in the Singapore National Cancer Registry, suggesting a low likelihood that any ethnic group was systematically excluded [28]. Also, approximately 1 out of 4 survivors would have been deterred if non-English tools were unavailable. The availability of service coordinators’ assistance for approximately 33% of responses obtained ensured that the model remained inclusive of survivors with poor literacy and technological knowledge. Adherence to screening did not differ by language choice and ethnicity, except for a 15% marginally lower completion rate among Malays than Chinese. This observation could be potentially explained by differences in perceived health status and health-seeking behaviour. Singapore-based studies revealed that Malays tend to report better self-rated health and lower adherence to cancer-related screening, reducing the perceived need for additional supportive services and screening for their cancer management [52,53]. Nevertheless, the overall general positive results on inclusivity underscored efforts to develop screening resources in multiple languages, multiple modes, and ensuring cultural diversity in the workforce.

Strategies targeting inclusivity further allowed the model to be flexible and adapt to restrictions imposed by the COVID-19 pandemic. Notably, service coordinators switched promptly to administer DTPL via phone calls, achieving >50% screening completion rates even during tightened restrictions. However, survivors without phone access likely remained underserved during COVID-19. Therefore, a hybrid mode comprising electronic platforms, phone calls, and in-person encounters should be adopted or preserved to maintain inclusivity and robustness to external changes such as the pandemic.

There were several areas for improvement in our care model. We encountered a significant barrier with screening mechanics and poor integration with electronic medical records, consistent with other centres’ experiences [54]. Extensive manpower utilized to support the manual components of screening incur significant opportunity costs that could potentially be reduced with digital solutions [55,56]. Coordinators could have played a more active role as health coaches or care navigators to improve outreach to underserved populations, such as the Malay group identified in our context, to further boost inclusivity [19,57]. Also, the COVID-19 pandemic compromised the model’s penetration due to deferred visits, failing to hit the expected caseload by almost 40% [58]. Lastly, community service referrals were impeded by a significant heterogeneity in referral workflows across community providers.

This study has several limitations. First, demographic and clinical characteristics of survivors who refused the model were not collected to reveal potential systematic differences. Second, the mode of screening tool completion served as a proxy for literacy as associated data were not routinely collected. Third, we could not ascertain how consistently screening results were discussed between oncologists and survivors. Fourth, we acknowledge potential limitations with the use of DT for screening, including the unknown optimal interval between repeated screening and unclear survivors’ perceptions of high distress [59]. Notwithstanding, the DT is brief for routine administration. Furthermore, the overall high positive and negative predictive values of >80% corresponding to the cut-off score employed strongly justify its usage [34]. Lastly, the evaluation of the implementation process was mainly conducted from a health system’s perspective. Thus, future work is underway to explore survivors’ and their caregivers’ experiences with the care model to supplement outcomes from this study. Additionally, the concurrent clinical trial as part of the hybrid study approach will elucidate the clinical impact of the model and facilitate subsequent economic evaluation to support long-term model adoption.

## Lessons learned

- Recognizing diverse care providers in integrated care provision, prospective adopters of a multidisciplinary supportive care model for cancer survivors in the outpatient setting should devote sufficient resources to engage the workforce continuously to sustain a positive implementation climate. Notably, a service coordinator role, preferably a person with health science-related background, should be carefully envisioned to complement the care team by providing general cancer survivors with navigation support or basic health coaching advice.
- A systematic approach should be adopted to facilitate access to community services and care providers from the tertiary settings. Foremost, mutually beneficial educational and outreach initiatives constitute a preliminary step to improve awareness of available community services and encourage collaboration opportunities. The derivation of decisive benefits from this interaction promotes mutualism to optimize the level of inter-organizational integration [60]. Community services should also be mapped to reported care needs in the integrated care pathways used in tertiary settings to encourage care coordination. A centralized referral system is further proposed to ensure care continuity through improved navigation and referral outcomes tracking.
- Key implementation strategies employed to ensure inclusivity of minority groups include developing screening resources in multiple languages, hybrid modes (electronic platforms, phone calls, and in-person encounters), and ensuring cultural diversity in the workforce. Further refinement of this approach should consider context-specific factors of the target setting and incorporate existing effective outreach strategies to minority groups.
- Inclusivity considerations provided additional positive impact as they allowed the care model to be flexible and remained robust to external changes such as the restrictions imposed by the COVID-19 pandemic. This flexibility is greatly enhanced by the inclusion of telehealth.

## Conclusion

Our multidisciplinary supportive care model designed with considerations of inclusivity and access to community services delivered integrated care to an ethnically diverse population. It demonstrated high fidelity, feasibility, and responsiveness to breast and gynaecological cancer survivors’ care needs in the outpatient setting. Community service referrals were facilitated systematically through the supportive care team and a hybrid screening approach preserved inclusivity while allowing flexible adaptations during COVID-19. Complementing ongoing research to evaluate the effectiveness of the model on survivor-level outcomes such as quality of life and symptom burden, future research should explore telehealth’s integration into supportive care models to streamline and promote integrated care. It would be useful to clarify how telehealth can further boost inclusivity and community engagement, providing access to multiple health disciplines and services through survivors’ preferred modes dynamically. Furthermore, the potential for telehealth to transform a multidisciplinary care team into an interdisciplinary one will be of great interest to advance integrated care provision.

## References

[1] NCI Dictionary of Cancer Terms [webpage on the internet]. [cited 2021]. Available from: https://www.cancer.gov/publications/dictionaries/cancer-terms/def/survivor.

[2] Multinational Association of Supportive Care in Cancer. Definition of supportive care [webpage on the internet]. [cited 2021; updated 2021]. Available from: https://www.mascc.org/mascc-strategic-plan.

[3] Hui D, Hoge G, Bruera E. Models of supportive care in oncology. Current opinion in oncology. 2021; 33(4): 259–66. DOI: 10.1097/CCO.0000000000000733 33720070 PMC8641044

[4] Fitch MI. Supportive care framework. Canadian Oncology Nursing Journal. 2008; 18(1): 6–14. DOI: 10.5737/1181912x181614 18512565

[5] Berman R, Davies A, Cooksley T, Gralla R, Carter L, Darlington E, et al. Supportive Care: An Indispensable Component of Modern Oncology. Clinical Oncology. 2020; 32(11): 781–8. DOI: 10.1016/j.clon.2020.07.020 32814649 PMC7428722

[6] Mollica MA, Mayer DK, Oeffinger KC, Kim Y, Buckenmaier SS, Sivaram S, et al. Follow-Up Care for Breast and Colorectal Cancer Across the Globe: Survey Findings From 27 Countries. JCO Glob Oncol. 2020; 6: 1394–411. DOI: 10.1200/GO.20.00180 32955943 PMC7529533

[7] Loh KW-J, Ng T, Choo SP, Saw HM, Mahendran R, Tan C, et al. Cancer Supportive and Survivorship Care in Singapore: Current Challenges and Future Outlook. Journal of Global Oncology. 2018; 4: 1–8. DOI: 10.1200/JGO.17.00117 PMC6223422 30241247

[8] Ng T, Toh MR, Cheung YT, Chan A. Follow-up care practices and barriers to breast cancer survivorship: perspectives from Asian oncology practitioners. Supportive Care in Cancer. 2015; 23(11): 3193–200. DOI: 10.1007/s00520-015-2700-2 25791392

[9] Institute of Medicine Committee on Psychosocial Services to Cancer Patients/Families in a Community Setting. The National Academies Collection: Reports funded by National Institutes of Health. In: Adler NE, Page AEK (eds.), Cancer Care for the Whole Patient: Meeting Psychosocial Health Needs. Washington, DC: National Academies Press (US) Copyright © 2008, National Academy of Sciences; 2008.

[10] Fagerlind H, Kettis A, Glimelius B, Ring L. Barriers against psychosocial communication: oncologists’ perceptions. J Clin Oncol. 2013; 31(30): 3815–22. DOI: 10.1200/JCO.2012.45.1609 24043746

[11] Chan RJ, Yates P, Li Q, Komatsu H, Lopez V, Thandar M, et al. Oncology practitioners’ perspectives and practice patterns of post-treatment cancer survivorship care in the Asia-Pacific region: results from the STEP study. BMC Cancer. 2017; 17(1): 715-. DOI: 10.1186/s12885-017-3733-3 29110686 PMC5674781

[12] Enthoven AC. Integrated delivery systems: the cure for fragmentation. Am J Manag Care. 2009; 15(10 Suppl): S284–90. 20088632

[13] Lee J, Johnson SR. ‘Fragmented’ cancer care. System is ‘chaotic, costly,’ IOM report says. Mod Healthc. 2013; 43(37): 8–9. 24195156

[14] Commission on Cancer. Optimal Resources for Cancer Care (2020 Standards) [webpage on the internet]. [cited 2021]. Available from: https://www.facs.org/-/media/files/quality-programs/cancer/coc/optimal_resources_for_cancer_care_2020_standards.ashx.

[15] van Nuenen FM, Donofrio SM, Tuinman MA, van de Wiel HBM, Hoekstra-Weebers JEHM. Feasibility of implementing the ‘Screening for Distress and Referral Need’ process in 23 Dutch hospitals. Supportive Care in Cancer. 2017; 25(1): 103–10. DOI: 10.1007/s00520-016-3387-8 27565789 PMC5127859

[16] Bultz BD, Groff SL, Fitch M, Blais MC, Howes J, Levy K, et al. Implementing screening for distress, the 6th vital sign: a Canadian strategy for changing practice. Psycho-Oncology. 2011; 20(5): 463–9. DOI: 10.1002/pon.1932 21456060

[17] Riba MB, Donovan KA, Andersen B, Braun I, Breitbart WS, Brewer BW, et al. Distress management, version 3.2019. JNCCN Journal of the National Comprehensive Cancer Network. 2019; 17(10): 1229–49. DOI: 10.6004/jnccn.2019.7358 31590149 PMC6907687

[18] Schouten B, Avau B, Bekkering GE, Vankrunkelsven P, Mebis J, Hellings J, et al. Systematic screening and assessment of psychosocial wellbeing and care needs of people with cancer. Cochrane Database of Systematic Reviews. 2019; 2019(3). DOI: 10.1002/14651858.CD012387.pub2 PMC6433560 30909317

[19] Deshields TL, Wells-Di Gregorio S, Flowers SR, Irwin KE, Nipp R, Padgett L, et al. Addressing distress management challenges: Recommendations from the consensus panel of the American Psychosocial Oncology Society and the Association of Oncology Social Work. CA: A Cancer Journal for Clinicians. 2021; caac.21672–caac. DOI: 10.3322/caac.21672 34028809

[20] El-Deiry WS, Giaccone G. Challenges in Diversity, Equity, and Inclusion in Research and Clinical Oncology. Front Oncol. 2021; 11: 642112. DOI: 10.3389/fonc.2021.642112 33842350 PMC8024634

[21] Jacobs LA, Shulman LN. Follow-up care of cancer survivors: challenges and solutions. The Lancet Oncology. 2017; 18(1): e19–e29. DOI: 10.1016/S1470-2045(16)30386-2 28049574

[22] Curran GM, Bauer M, Mittman B, Pyne JM, Stetler C. Effectiveness-implementation Hybrid Designs. Medical Care. 2012; 50(3): 217–26. DOI: 10.1097/MLR.0b013e3182408812 22310560 PMC3731143

[23] Jacobsen PB, Norton WE. The role of implementation science in improving distress assessment and management in oncology: a commentary on “Screening for psychosocial distress among patients with cancer: implications for clinical practice, healthcare policy, and dissemination to enhance cancer survivorship”. Transl Behav Med. 2019; 9(2): 292–5. DOI: 10.1093/tbm/ibz022 30870569 PMC6610164

[24] McCarter K, Fradgley EA, Britton B, Tait J, Paul C. Not seeing the forest for the trees: a systematic review of comprehensive distress management programs and implementation strategies. Current opinion in supportive and palliative care. 2020; 14(3): 220–31. DOI: 10.1097/SPC.0000000000000513 32657813

[25] Pinnock H, Barwick M, Carpenter CR, Eldridge S, Grandes G, Griffiths CJ, et al. Standards for Reporting Implementation Studies (StaRI) Statement. BMJ (Online). 2017; 356(March): 1–9. DOI: 10.1136/bmj.i6795 PMC5421438 28264797

[26] National Cancer Centre Singapore – About Us [webpage on the internet]. [cited 2021]. Available from: https://www.nccs.com.sg/giving/about-us.

[27] Wong L. New $2.1-million cancer care programme to support 4,000 breast cancer survivors [webpage on the internet]. [2021]; [updated 2019]. Available from: https://www.straitstimes.com/singapore/health/new-21-million-cancer-care-programme-to-support-4000-breast-cancer-survivors.

[28] Singapore Cancer Registry Annual Report 2018 [webpage on the internet]. [cited 2021]. Available from: https://www.nrdo.gov.sg/docs/librariesprovider3/default-document-library/scr-annual-report-2018.pdf?sfvrsn=bcf56c25_0.

[29] Damschroder LJ, Aron DC, Keith RE, Kirsh SR, Alexander JA, Lowery JC. Fostering implementation of health services research findings into practice: a consolidated framework for advancing implementation science. Implementation Science. 2009; 4(1): 50–. DOI: 10.1186/1748-5908-4-50 19664226 PMC2736161

[30] Proctor EK, Powell BJ, McMillen JC. Implementation strategies: recommendations for specifying and reporting. Implement Sci. 2013; 8: 139. DOI: 10.1186/1748-5908-8-139 24289295 PMC3882890

[31] Powell BJ, Waltz TJ, Chinman MJ, Damschroder LJ, Smith JL, Matthieu MM, et al. A refined compilation of implementation strategies: results from the Expert Recommendations for Implementing Change (ERIC) project. Implement Sci. 2015; 10: 21. DOI: 10.1186/s13012-015-0209-1 25889199 PMC4328074

[32] National Cancer Centre Singapore. Coping with Cancer and Treatments [webpage on the internet]. [cited 2021]. Available from: https://www.nccs.com.sg/patient-care/pages/coping-with-cancer-and-treatments.aspx.

[33] BorjAlilu S, Kaviani A, Helmi S, Karbakhsh M, Mazaheri MA. Exploring the Role of Self-Efficacy for Coping With Breast Cancer: A Systematic Review. Archives of Breast Cancer. 2017; 4(2): 42–57.

[34] Lim HA, Mahendran R, Chua J, Peh C-X, Lim S-E, Kua E-H. The Distress Thermometer as an ultra-short screening tool: A first validation study for mixed-cancer outpatients in Singapore. Comprehensive Psychiatry. 2014; 55(4): 1055–62. DOI: 10.1016/j.comppsych.2014.01.008 24556515

[35] Chua AQ, Tan MMJ, Verma M, Han EKL, Hsu LY, Cook AR, et al. Health system resilience in managing the COVID-19 pandemic: lessons from Singapore. BMJ Global Health. 2020; 5(9): e003317–e. DOI: 10.1136/bmjgh-2020-003317 PMC7496566 32938609

[36] Chen JIP, Yap JC-H, Hsu LY, Teo YY. COVID-19 and Singapore: From Early Response to Circuit Breaker. Annals of the Academy of Medicine, Singapore. 2020; 49(8): 561–72. DOI: 10.47102/annals-acadmedsg.2020239 33164026

[37] Proctor E, Silmere H, Raghavan R, Hovmand P, Aarons G, Bunger A, et al. Outcomes for Implementation Research: Conceptual Distinctions, Measurement Challenges, and Research Agenda. Administration and Policy in Mental Health and Mental Health Services Research. 2011; 38(2): 65–76. DOI: 10.1007/s10488-010-0319-7 20957426 PMC3068522

[38] Zebrack B, Kayser K, Bybee D, Padgett L, Sundstrom L, Jobin C, et al. A practice-based evaluation of distress screening protocol adherence and medical service utilization. JNCCN Journal of the National Comprehensive Cancer Network. 2017; 15(7): 903–12. DOI: 10.6004/jnccn.2017.0120 28687578

[39] Jacobsen PB, Shibata D, Siegel EM, Lee J-H, Fulp WJ, Alemany C, et al. Evaluating the quality of psychosocial care in outpatient medical oncology settings using performance indicators. Psycho-Oncology. 2011; 20(11): 1221–7. DOI: 10.1002/pon.1849 20878724 PMC4497369

[40] Zebrack B, Kayser K, Sundstrom L, Savas SA, Henrickson C, Acquati C, et al. Psychosocial Distress Screening Implementation in Cancer Care: An Analysis of Adherence, Responsiveness, and Acceptability. Journal of Clinical Oncology. 2015; 33(10): 1165–70. DOI: 10.1200/JCO.2014.57.4020 25713427

[41] Shimizu K, Ishibashi Y, Umezawa S, Izumi H, Akizuki N, Ogawa A, et al. Feasibility and usefulness of the ‘Distress Screening Program in Ambulatory Care’ in clinical oncology practice. Psycho-Oncology. 2010; 19(7): 718–25. DOI: 10.1002/pon.1616 19673010

[42] McLeod DL, Morck AC, Curran JA. A pan-Canadian web-based education program to support screening for distress: Evaluation of outcomes. Palliative and Supportive Care. 2014; 12(1): 15–23. DOI: 10.1017/S1478951513000072 23942172

[43] Grassi L, Rossi E, Caruso R, Nanni MG, Pedrazzi S, Sofritti S, et al. Educational intervention in cancer outpatient clinics on routine screening for emotional distress: an observational study. Psycho-Oncology. 2011; 20(6): 669–74. DOI: 10.1002/pon.1944 21370316

[44] Nguyen H, Butow P, Dhillon H, Sundaresan P. A review of the barriers to using Patient-Reported Outcomes (PROs) and Patient-Reported Outcome Measures (PROMs) in routine cancer care. Journal of Medical Radiation Sciences. 2021; 68(2): 186–95. DOI: 10.1002/jmrs.421 32815314 PMC8168064

[45] Sandhu S, King Z, Wong M, Bissell S, Sperling J, Gray M, et al. Implementation of Electronic Patient-Reported Outcomes in Routine Cancer Care at an Academic Center: Identifying Opportunities and Challenges. JCO Oncology Practice. 2020; 16(11): e1255–e63. DOI: 10.1200/OP.20.00357 32926662

[46] Hamilton J, Kroska EB. Distress predicts utilization of psychosocial health services in oncology patients. Psycho-Oncology. 2019; 28(1): 61–7. DOI: 10.1002/pon.4910 30286522

[47] Tondorf T, Grossert A, Rothschild SI, Koller MT, Rochlitz C, Kiss A, et al. Focusing on cancer patients’ intentions to use psychooncological support: A longitudinal, mixed-methods study. Psycho-Oncology. 2018; 27(6): 1656–63. DOI: 10.1002/pon.4735 29656415 PMC6001470

[48] Ploos van Amstel FK, Peters MEWJ, Donders R, Schlooz-Vries MS, Polman LJM, Graaf WTA, et al. Does a regular nurse-led distress screening and discussion improve quality of life of breast cancer patients treated with curative intent? A randomized controlled trial. Psycho-Oncology. 2020; 29(4): 719–28. DOI: 10.1002/pon.5324 31876036

[49] Frey Nascimento A, Tondorf T, Rothschild SI, Koller MT, Rochlitz C, Kiss A, et al. Oncologist recommendation matters!—Predictors of psycho-oncological service uptake in oncology outpatients. Psycho-Oncology. 2019; 28(2): 351–7. DOI: 10.1002/pon.4948 30466146

[50] Opening Address By Dr Amy Khor, Senior Minister Of State, Ministry Of Health, At The Singapore Healthcare Management Congress 2018, 14 August 2018 [webpage on the internet]. [cited 2021]. Available from: https://www.moh.gov.sg/news-highlights/details/opening-address-by-dr-amy-khor-senior-minister-of-state-ministry-of-health-at-the-singapore-healthcare-management-congress-2018-14-august-2018. DOI: 10.1200/JCO.2015.64.3809

[51] Runowicz CD, Leach CR, Henry NL, Henry KS, Mackey HT, Cowens-Alvarado RL, et al. American Cancer Society/American Society of Clinical Oncology Breast Cancer Survivorship Care Guideline. Journal of Clinical Oncology. 2016; 34(6): 611–35. DOI: 10.1200/JCO.2015.64.3809 26644543

[52] Chan TK-C, Tan LWL, van Dam RM, Seow WJ. Cancer Screening Knowledge and Behavior in a Multi-Ethnic Asian Population: The Singapore Community Health Study. Frontiers in Oncology. 2021; 11. DOI: 10.3389/fonc.2021.684917 PMC8406849 34476210

[53] Lim W-Y, Ma S, Heng D, Bhalla V, Chew SK. Gender, ethnicity, health behaviour & self-rated health in Singapore. BMC Public Health. 2007; 7(1): 184–. DOI: 10.1186/1471-2458-7-184 17655774 PMC1976324

[54] Knies AK, Jutagir DR, Ercolano E, Pasacreta N, Lazenby M, McCorkle R. Barriers and facilitators to implementing the commission on cancer’s distress screening program standard. Palliative and Supportive Care. 2019; 17(03): 253–61. DOI: 10.1017/S1478951518000378 29880068 PMC6286692

[55] Wu AW, Kharrazi H, Boulware LE, Snyder CF. Measure once, cut twice—adding patient-reported outcome measures to the electronic health record for comparative effectiveness research. Journal of Clinical Epidemiology. 2013; 66(8): S12–S20. DOI: 10.1016/j.jclinepi.2013.04.005 23849145 PMC3779680

[56] Jensen RE, Snyder CF, Abernethy AP, Basch E, Potosky AL, Roberts AC, et al. Review of Electronic Patient-Reported Outcomes Systems Used in Cancer Clinical Care. Journal of Oncology Practice. 2014; 10(4): e215–e22. DOI: 10.1200/JOP.2013.001067 24301843 PMC4094646

[57] Paskett ED, Harrop JP, Wells KJ. Patient navigation: An update on the state of the science. CA: A Cancer Journal for Clinicians. 2011; 61(4): 237–49. DOI: 10.3322/caac.20111 21659419 PMC3623288

[58] Kanesvaran R, Chia CS, Yap SP, Wang MLC, Tham CK, Lim ST, et al. Cancer Versus COVID-19: A Coordinated Disease Outbreak Response System (DORS) to Combat COVID-19 at the National Cancer Centre Singapore. Annals of the Academy of Medicine, Singapore. 2020; 49(10): 807–9. DOI: 10.47102/annals-acadmedsg.2020291 33283845

[59] VanHoose L, Black LL, Doty K, Sabata D, Twumasi-Ankrah P, Taylor S, et al. An analysis of the distress thermometer problem list and distress in patients with cancer. Support Care Cancer. 2015; 23(5): 1225–32. DOI: 10.1007/s00520-014-2471-1 25315367

[60] Ahgren B. The art of integrating care: theories revisited. The Open Public Health Journal. 2012; 5(1). DOI: 10.2174/1874944501205010036

